# Inhibition of Autotaxin and Lysophosphatidic Acid Receptor 5 Attenuates Neuroinflammation in LPS-Activated BV-2 Microglia and a Mouse Endotoxemia Model

**DOI:** 10.3390/ijms22168519

**Published:** 2021-08-07

**Authors:** Lisha Joshi, Ioanna Plastira, Eva Bernhart, Helga Reicher, Alexander Triebl, Harald C. Köfeler, Wolfgang Sattler

**Affiliations:** 1 Division of Molecular Biology and Biochemistry, Gottfried Schatz Research Center, Medical University of Graz, 8010 Graz, Austria; lisha.joshi@medunigraz.at (L.J.); ioanna.plastira@medunigraz.at (I.P.); eva.bernhart@medunigraz.at (E.B.); helga.reicher@medunigraz.at (H.R.); 2 Core Facility Mass Spectrometry, Medical University of Graz, 8010 Graz, Austria; alex.triebl@gmx.at (A.T.); harald.koefeler@medunigraz.at (H.C.K.); 3 BioTechMed Graz, 8010 Graz, Austria

**Keywords:** AS2717638, chemokines, cytokines, neurotoxicity, PF8380

## Abstract

Increasing evidence suggests that systemic inflammation triggers a neuroinflammatory response that involves sustained microglia activation. This response has deleterious consequences on memory and learning capability in experimental animal models and in patients. However, the mechanisms connecting systemic inflammation and microglia activation remain poorly understood. Here, we identify the autotaxin (ATX)/lysophosphatidic acid (LPA)/LPA-receptor axis as a potential pharmacological target to modulate the LPS-mediated neuroinflammatory response in vitro (the murine BV-2 microglia cell line) and in vivo (C57BL/6J mice receiving a single i.p. LPS injection). In LPS-stimulated (20 ng/mL) BV-2 cells, we observed increased phosphorylation of transcription factors (STAT1, p65, and c-Jun) that are known to induce a proinflammatory microglia phenotype. LPS upregulated ATX, TLR4, and COX2 expression, amplified NO production, increased neurotoxicity of microglia conditioned medium, and augmented cyto-/chemokine concentrations in the cellular supernatants. PF8380 (a type I ATX inhibitor, used at 10 and 1 µM) and AS2717638 (an LPA5 antagonist, used at 1 and 0.1 µM) attenuated these proinflammatory responses, at non-toxic concentrations, in BV-2 cells. In vivo, we demonstrate accumulation of PF8380 in the mouse brain and an accompanying decrease in LPA concentrations. In vivo, co-injection of LPS (5 mg/kg body weight) and PF8380 (30 mg/kg body weight), or LPS/AS2717638 (10 mg/kg body weight), significantly attenuated LPS-induced iNOS, TNFα, IL-1β, IL-6, and CXCL2 mRNA expression in the mouse brain. On the protein level, PF8380 and AS2717638 significantly reduced TLR4, Iba1, GFAP and COX2 expression, as compared to LPS-only injected animals. In terms of the communication between systemic inflammation and neuroinflammation, both inhibitors significantly attenuated LPS-mediated systemic TNFα and IL-6 synthesis, while IL-1β was only reduced by PF8380. Inhibition of ATX and LPA5 may thus provide an opportunity to protect the brain from the toxic effects that are provoked by systemic endotoxemia.

## 1. Introduction

Systemic, LPS-induced inflammation activates an inflammatory response in the brain that involves the microglia, which are the resident immune cells of the central nervous system (CNS; [1,2]). These tissue-resident macrophages represent a first line of defense against infection and tissue injury. Resting microglia are in a constant surveillance state, and their processes continuously palpate the surface of neighboring cells and the parenchyma, to scan the homeostasis of the microenvironment [3]. A specific set of receptors, termed the ‘microglia sensome’, are expressed by microglia, to support their function [4]. These receptors interact with pathogens or exogenous ligands, thereby providing a signaling platform that induces different activation/polarization programs of the microglia [5].

The microglia play an important role in communicating between systemic inflammation and the CNS. This is supported by a growing body of evidence demonstrating that microglia activation and neuroinflammation are induced by systemic inflammatory events [6,7]. The experimental animal models that are used to study neuroinflammation include single or multiple i.p. injections of LPS from E.coli, to trigger systemic inflammation [8]. Peripheral administration of LPS in mice is accompanied by astrocyte and microglia activation, COX2 and iNOS expression, as well as increased cytokine and chemokine expression in the brain, and is therefore commonly used as an experimental model to induce neuroinflammation [9]. Activated microglia are able to release inflammatory and neurotoxic factors, and an unresolved microglia response can result in neuronal cell death and/or induce neurodegenerative disease [6]. Whether or not systemic LPS enters the brain is not entirely clear; Banks and Robinson reported minimal blood–brain barrier (BBB) permeability for intravenously injected ^125^I-labeled LPS in mice [10], while Vargas-Caraveo and colleagues reported LPS infiltration into the rat brain via a lipoprotein-mediated mechanism [11]. In addition, systemically produced cytokines and/or lipid mediators can communicate with the brain via neural, humoral, and brain endothelial cell-mediated pathways, to induce microglia activation [12,13].

Several subsets of receptor families contribute to microglia activation in response to inflammatory stimuli. These include Toll-like receptors, pattern recognition receptors, and several cyto-/chemokine receptors [14]. In addition, microglia activation can be induced by a specific subset of G-protein-coupled receptors, namely, the lysophosphatidic acid receptor (LPAR) family that consists of six members, termed LPA1-6 [15,16]. The signaling outcome is determined by the structure of the LPA ligand and G-protein coupling [17] that drives diverse physiological and pathophysiological processes in the brain [15]. Although several pathways can contribute to LPA synthesis, the autotaxin (ATX; gene name Enpp2) pathway is considered to be the quantitatively most important, since mice that are heterozygous for Enpp2 produce only 50% of LPA as compared to wild-type animals [18]. During development, LPA signaling is essential for normal neurogenesis and function [15], and LPA is detectable in the embryonic brain, choroid plexus, meninges, neural tube, blood vessels, spinal cord, and cerebrospinal fluid (CSF), at nanomolar to low micromolar concentrations [15]. However, in response to injury, LPA concentrations increase in the brain and CSF [19,20,21,22,23], and aberrant signaling can contribute to multiple neuropathological disorders, including neuroinflammation following stroke and cerebral edema [24].

In isolated primary murine neonatal microglia and the murine BV-2 microglia cell line, exogenous LPA induces a pro-inflammatory phenotype [25], and many of these inflammatory reactions are attenuated in the presence of LPA5 antagonists [26]. When BV-2 microglia are polarized with LPS, they increase ATX transcription and LPA secretion, along with the release of proinflammatory cytokines, while IL-10 expression is decreased [27]. However, in response to ATX overexpression, BV-2 cells secrete significantly higher basal IL-10 concentrations that provide partial protection against an LPS-induced inflammatory phenotype [27]. In vivo, induction of acute and chronic inflammatory conditions in mice, by LPS, resulted in significantly increased LPA concentrations and differential regulation of LPAR and ATX gene expression in the brain and FACS-sorted microglia [25]. Building on these previous findings, the present study aimed to clarify whether pharmacological inhibitors of ATX (PF8380) and LPA5 (AS2717638) would i) dampen the proinflammatory reaction of LPS-primed microglia in vitro (BV-2 cells), and ii) attenuate the LPS-induced neuroinflammatory response in the mouse brain in vivo.

## 2. Results

**Figure 1:**
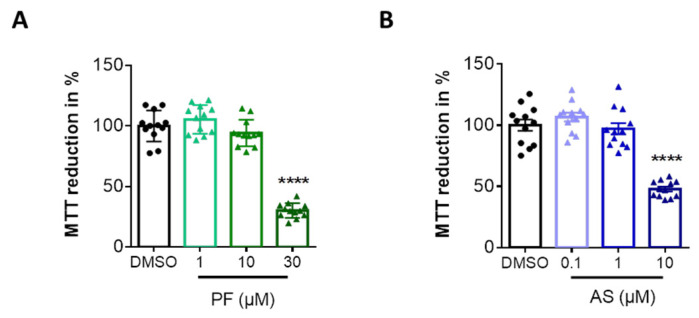
Evaluation of cytotoxicity of PF8380 and AS2717638 in BV-2 microglia**.** Cells were treated with increasing concentrations of (**A**) the ATX inhibitor PF8380 (‘PF’) and (**B**) the LPA5 inhibitor AS2717638 (‘AS’) for 24 h. Dimethylsulfoxide (DMSO) was used as vehicle control. Dose-dependent effect of the inhibitors on MTT reduction in cells was compared to control. Results are shown as mean ± SEM of three independent experiments. (**** *p* < 0.0001, compared to control; one-way ANOVA with Bonferroni correction).

In the first series of experiments, we determined the range of non-toxic concentrations for the two pharmacological inhibitors (PF8380 as ATX, and AS2717638 as LPA5 antagonist; [28,29]) that were used throughout our study. Incubation of BV-2 cells with increasing concentrations of PF8380 indicated a decrease in MTT reduction, by 70% at the highest concentration used (30 µM, 24 h; Figure 1A). AS2717638 was without effect on MTT reduction at 0.1 and 1 µM, but compromised cell viability by 50% when used at 10 µM for 24 h (Figure 1B). For these reasons, PF8380 was used at 1 and 10 µM, while AS2717638 was used at 0.1 and 1 µM during the experiments that are described below.

**Figure 2:**
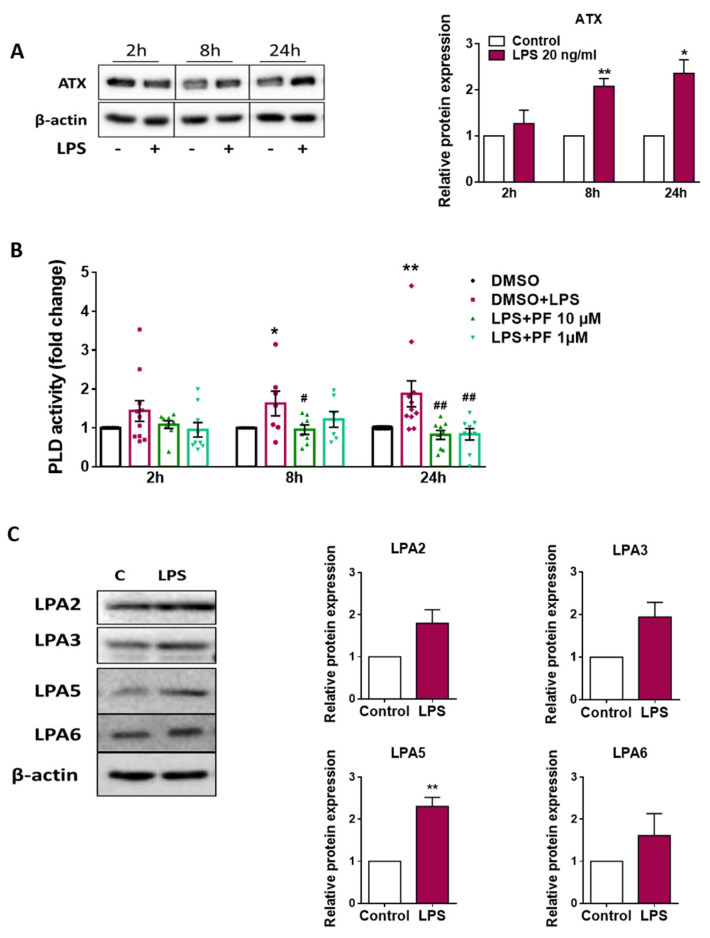
Effect of LPS on ATX protein expression, PLD activity and LPA receptor expression in BV-2 microglia**.** (**A**) Cells were treated in the absence (‘-‘) or presence (‘+’) of LPS (20 ng/mL) for the indicated times. Cell protein lysates were collected and ATX was detected by immunoblotting. β-actin was used as loading control. One representative blot of ATX is shown. Densitometric evaluation of immunoreactive bands is shown in the bar graphs. Values are expressed as mean ± SEM of three independent experiments. (* *p* < 0.05, ** *p* < 0.01 compared to control; unpaired Student’s t test). (**B**) Cells were treated with LPS (20 ng/mL) in the absence or presence of PF8380 (‘PF’; 10 and 1 µM) for the indicated times. DMSO was used as vehicle control. PLD activity in the supernatant was measured by phospholipase D kit (Sigma) and compared with their appropriate control. Results are presented as mean ± SEM of three independent experiments. (* *p* < 0.05, ** *p* < 0.01; # *p* < 0.05, ## *p* < 0.01 compared to LPS-treated cells; one-way ANOVA with Bonferroni correction). (**C**) Cells were incubated with LPS (20 ng/mL) for 24 h. Expression of LPA2, -3, -5 and -6 was detected by immunoblotting. β-actin was used as loading control. One representative blot for each protein is shown. Densitometric evaluation of immunoreactive bands is shown in the bar graphs. Values are expressed as mean ± SEM of three independent experiments. (** *p* < 0.01 compared to control; unpaired Student’s *t* test).

To get an indication about the effects of LPS on ATX expression, we performed Western blot experiments. In unstimulated cells, the intensity of the ATX band slightly decreased at 8 and 24 h, when compared to 2 h. When compared to unstimulated cells, LPS significantly increased the ATX protein by about two-fold at 8 and 24 h (Figure 2A; bar graphs in the right panel represent densitometric evaluation of the ATX bands). ATX hydrolyzes LPC to produce LPA. To test the effect of LPS on ATX activity, and to determine the efficacy of PF8380, we evaluated phospholipase D (PLD) activity as a surrogate readout for ATX activity in supernatants that were collected from DMSO (vehicle for PF8380), LPS- and LPS/PF8380-treated cells. These data indicate that LPS treatment significantly increased PLD activity in the supernatants (up to two-fold; Figure 2B). PF8380 (10 and 1 µM) treatment significantly attenuated LPS-induced PLD activity.

Next, we studied the impact of LPS on LPAR expression in BV-2 cells by Western blotting (representative blots are shown in Figure 2C). These studies revealed that BV-2 cells express LPA2, -3, -5, and -6 immunoreactive protein, while LPA1 and -4 were undetectable (as observed previously by qPCR analyses; [30]). The bar graphs display relative protein expression of the individual LPA receptors in untreated or LPS-treated (20 ng/mL for 24 h) BV-2 cells. Under the experimental conditions that were employed here, LPS treatment tended to upregulate LPA receptor expression, and this reached statistical significance (2.2-fold up) for LPA5 (Figure 2C).

**Figure 3:**
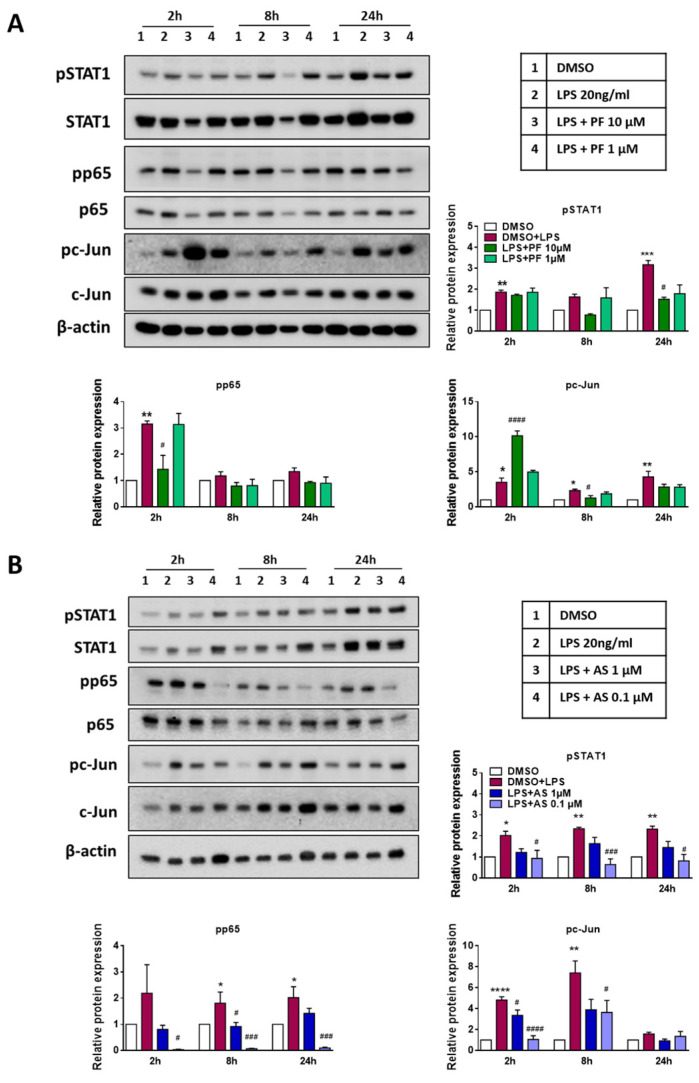
Inhibition of ATX and LPA5 regulates LPS-induced transcription factor phosphorylation**.** Cells were treated with DMSO (vehicle control), LPS (20 ng/mL) plus DMSO, and (**A**) LPS plus PF8380 (‘PF’; 10 and 1 µM) or (**B**) LPS plus AS2717638 (‘AS’; 1 and 0.1 µM) for the indicated times. Cell lysate were collected and protein expression of phosphorylated STAT1, p65, and c-Jun along with their total protein was monitored using immunoblotting. β-actin was used as loading control. One representative blot for each protein is shown. Densitometric evaluation of immunoreactive bands is shown in the bar graphs. Values are expressed as mean ± SEM of three independent experiments. (* *p* < 0.05, ** *p* < 0.01, *** *p* < 0.001, **** *p* < 0.0001 compared to DMSO control; # *p* < 0.05, ### *p* < 0.001, #### *p* < 0.0001 compared to LPS-treated cells; one-way ANOVA with Bonferroni correction).

In the next set of experiments, we analyzed the phosphorylation status of transcription factors (TF) that are known to induce a proinflammatory microglia phenotype that is characterized by the induction of COX-2, NO and ROS production, and the secretion of proinflammatory cyto-/chemokines [31]. To investigate a potential role of ATX and LPA5 in this scenario, BV-2 cells were activated by LPS in the absence or presence of PF8380 and AS2717638, followed by Western blot analysis. In response to LPS, phosphorylation of STAT1, p65, and c-Jun was significantly increased at one or more time points (Figure 3; bar graphs represent densitometric evaluation; the table inset describes the four treatment conditions, as indicated above the Western blots). The co-incubation of BV-2 cells with LPS/PF8380 (Figure 3A) significantly decreased STAT1 (24 h), p65 (2 h), and c-Jun (8 h) phosphorylation. The 10-fold increase in c-Jun phosphorylation, in response to PF8380 (10 µM) at 2 h, was unexpected, and currently we cannot offer a plausible explanation for this observation. 

A similar strategy was used to investigate the effect of the LPA5 antagonist AS2717638 (Figure 3B). The LPA5 antagonist effectively suppressed phosphorylation of STAT1, p65, and c-Jun, at one or more time points. Interestingly, in these experiments, the lower concentration (0.1 µM) had (for yet unknown reasons) a more pronounced inhibitory potential as compared to the higher concentration (1 µM).

**Figure 4:**
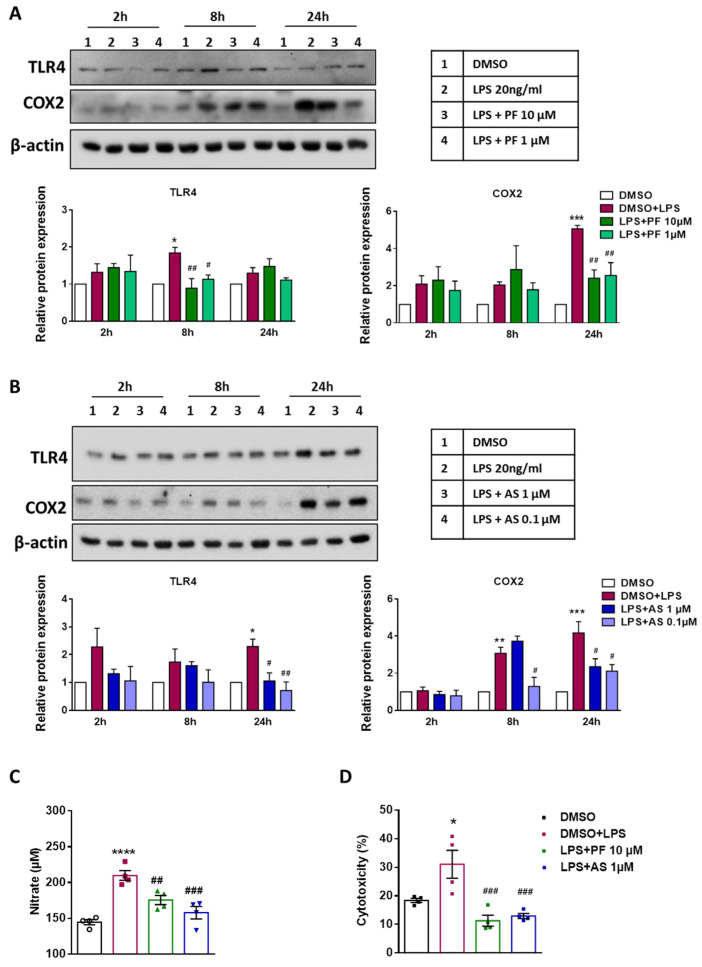
Inhibition of ATX and LPA5 down-regulates LPS-induced TLR4, COX-2 expression, nitric oxide production in BV-2 cells and reduces neurotoxicity of BV-2-conditioned medium. Cells were treated with DMSO (vehicle control), LPS (20 ng/mL) plus DMSO, (**A**) LPS plus PF8380 (‘PF’; 10 and 1 µM) or (**B**) LPS plus AS2717638 (‘AS’; 1 and 0.1 µM) for the indicated times. Cell lysates were collected and protein expression of TLR4 and COX2 was monitored by immunoblotting. β-actin was used as loading control. One representative blot for each protein is shown. Densitometric evaluation of immunoreactive bands is shown in the bar graphs. (**C**) Cells were treated with DMSO, LPS plus DMSO, and LPS plus PF8380 (‘PF’; 10 µM) or LPS plus AS2717638 (‘AS’; 1 µM) for 24 h. The production of NO was determined by measuring the total nitrate concentration in the supernatants. (**D**) CATH.a neurons were incubated for 24 h with conditioned media collected from LPS-treated (in the absence or presence of PF8380 (‘PF’; 10 µM) or AS2717638 (‘AS’; 1 µM)) BV-2 cells for 24 h. LDH levels were detected and neurotoxixity was calculated according to manufacturer’s protocol. Values are expressed as mean ± SEM of three independent experiments. (* *p* < 0.05, ** *p* < 0.01, *** *p* < 0.001, **** *p* < 0.0001 compared to DMSO control; # *p* < 0.05, ## *p* < 0.01, ### *p*< 0.001 compared to LPS-treated cells; one-way ANOVA with Bonferroni correction).

We then moved on to analyze the expression of a selected set of proteins that are known targets of these proinflammatory TF. To investigate if the ATX and LPA5 antagonists impact TLR4 receptor expression in LPS-challanged cells, Western blot analyses were performed. These experiments showed that LPS induced TLR4 at 8 h and 24 h (about two-fold) in BV-2 cells, while PF8380 treatment suppressed this increase (Figure 4A). LPS also significantly increased the expression of COX2 at 24 h and this effect was abrogated by PF8380 (Figure 4A). Qualitatively similar results were obtained in a second set of experiments that were performed in the absence or presence of AS2717638; LPS induced TLR4 and COX2 (Figure 4B; bar graphs represent densitometric evaluation), while immunoreactive TLR4 and COX2 levels were attenuated by LPA5 inhibition. 

We further examined the impact of PF8380 and AS2717638 on nitric oxide (NO) production in LPS-treated BV-2 cells. LPS increased NO production (detected as nitrate), and both ATX and LPA5 inhibition reduced nitrate levels (Figure 4C). To determine potential neurototoxic properties of the LPS-induced secretome of BV-2 cells, murine CATH.a neurons were incubated with supernatants that were collected from LPS- or LPS/inhibitor-treated BV-2 cells. These experiments revealed significant neurotoxicity of the media that were collected from LPS-activated cells (Figure 4D). In contrast, the pre-conditioned medium that was obtained from LPS/inhibitor-treated cells did not affect neuronal viability, and induced a slightly lower lactate dehydrogenase (LDH) release from the neuron cultures as compared to the medium that was collected from the untreated cells (Figure 4D).

**Figure 5:**
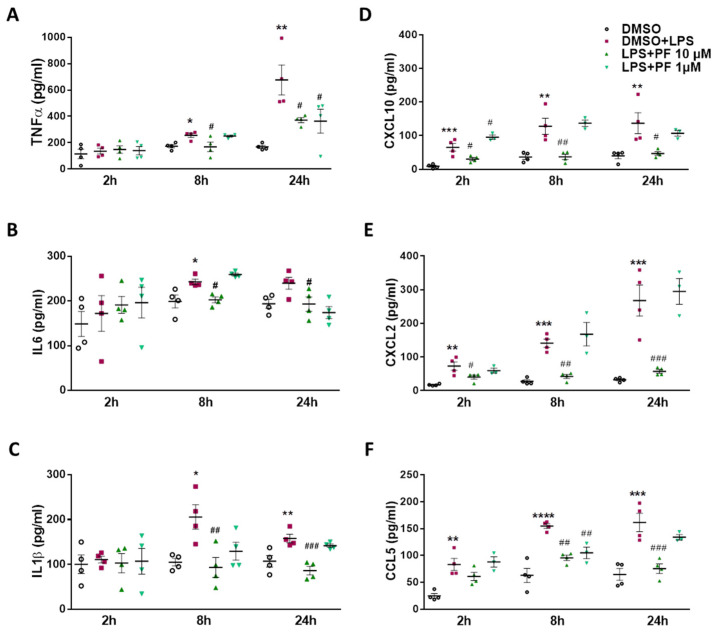
Inhibition of ATX by PF8380 attenuates LPS-induced secretion of cyto-/chemokines in BV-2 microglia. Cells were treated with DMSO control, LPS (20 ng/mL) plus DMSO in the absence or presence of PF8380 (‘PF’; 10 and 1 µM) for the indicated times. Supernatants were collected. (**A**–**F**) Concentration of cytokines TNFα, IL6, and IL-1β and chemokines CXCL10, CXCL2, and CCL5 were quantified by ELISA. Values are expressed as mean ± SEM of three independent experiments. (* *p* < 0.05, ** *p* < 0.01, *** *p* < 0.001, **** *p* < 0.0001 compared to DMSO control; # *p* < 0.05, ## *p* < 0.01, ### *p* < 0.001 compared to LPS-treated cells; one-way ANOVA with Bonferroni correction).

**Figure 6:**
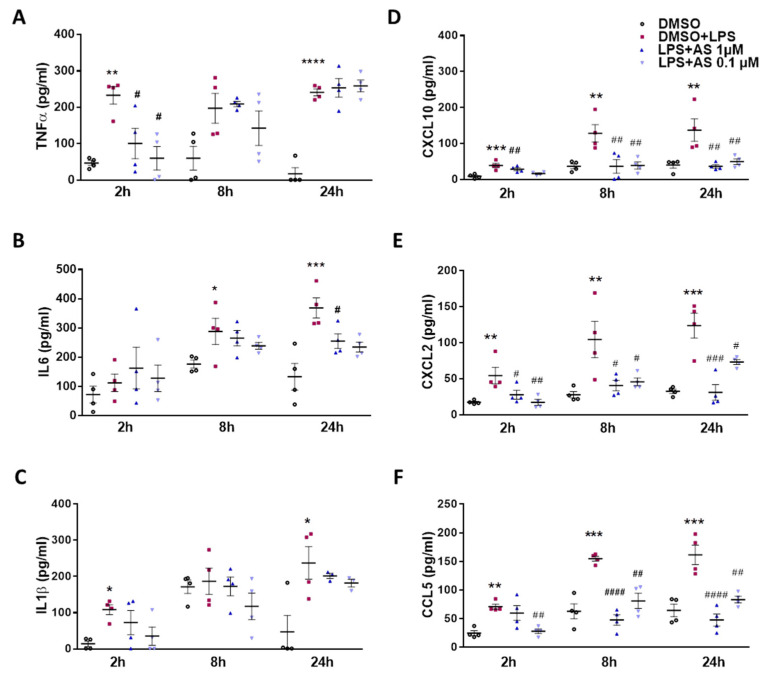
Inhibition of LPA5 by AS2717638 attenuates LPS-induced secretion of cyto-/chemokines in BV-2 microglia. Cells were treated with DMSO control, LPS plus DMSO in the absence or presence of AS2717638 (‘AS’; 1 and 0.1 µM) for the indicated times. Supernatants were collected. (**A–F**) Concentration of cytokines TNFα, IL6, and IL-1β and chemokines CXCL10, CXCL2, and CCL5 were quantified by ELISA. Values are expressed as mean ± SEM of three independent experiments. (* *p* < 0.05, ** *p* < 0.01, *** *p* < 0.001, **** *p* < 0.0001 compared to DMSO control; # *p* < 0.05, ## *p* < 0.01, ### *p* < 0.001, #### *p* < 0.0001 compared to LPS-treated cells; one-way ANOVA with Bonferroni correction).

Next, we studied cyto-/chemokine secretion from LPS-stimulated BV-2 cells and the impact of the two inhibitors on extracellular accumulation of these analytes. LPS significantly elevated TNFα concentrations at 8 and 24 h, and this increase was significantly reduced by 10 µM PF8380 at 8 and 24 h (Figure 5A). Comparable observations were made for IL6 (Figure 5B) and IL-1β (Figure 5C) at 8 and/or 24 h. The results for chemokine secretion (CXCL10, CXCL2, and CCL5; Figure 5D–F) were more clear-cut, as follows: LPS significantly increased extracellular accumulation of the three chemokines at all time points and in the presence of the ATX inhibitor (10 µM); this effect was significantly attenuated at 2, 8, and 24 h post LPS addition (Figure 5D–F). The LPA5 inhibitor decreased TNFα secretion at 2 h (Figure 6A), inhibited IL-6 at 24 h (Figure 6B), and was without effect on IL-1β release (Figure 6C). In line with the effects observed in Figure 5, the LPA5 inhibitor significantly reduced chemokine secretion at 0.1 and 1 µM at all the time points studied (Figure 6D–F).

**Figure 7:**
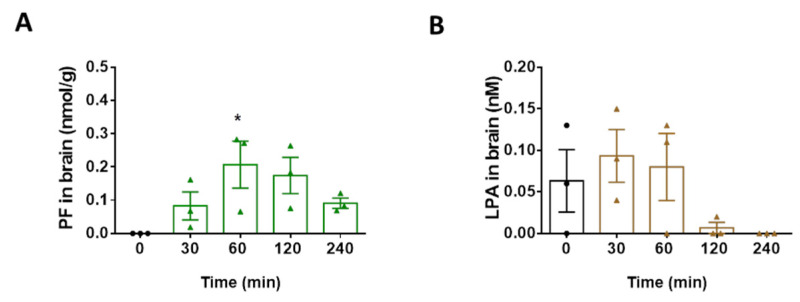
PF8380 crosses the blood–brain barrier and attenuates LPA levels in mouse brain. Animals received PF8380 (‘PF’) dissolved in oral formulation vehicle at a dose of 30 mg/kg by gavage to get an indication about oral bioavailability. At the indicated times, mice were sacrificed, perfused, and brains were dissected and snap frozen. The tissue homogenates were extracted using a modified Bligh & Dyer HCl method. Quantification of (**A**) PF8380 (‘PF’) and (**B**) LPA in brain was performed by LC-MS/MS. Results are presented as mean values ± SEM of three mice per group (* *p* < 0.05 compared to control; one-way ANOVA with Bonferroni correction).

We then asked whether PF8380 and AS1727638 would beneficially affect neuroinflammatory parameters in vivo, in a murine endotoxemia model. One of the main criteria for effective neurotherapeutic agents is their ability to cross the BBB. In the first step, we determined the BBB permeability of PF8380 in C57BL/6 mice. PF8380 was administered as a single dose (900 µg in 450 µL vehicle) by gavage. At the indicated time points, the animals (n = 3) were anesthetized with 150 mg/kg pentobarbital, and transcardially perfused with PBS. Subsequently, the brains were removed, extracted, and PF8380 and LPA concentrations were quantitated by LC-MS/MS, using external calibration [32]. The maximal PF8380 concentration was observed 60 min post application (0.21 ± 0.122 nmoles/g brain; Figure 7A). In PF8380-injected animals, the brain LPA levels dropped to concentrations below the baseline at 120 min post application (Figure 7B). Transport of the AS2717638 compound across the BBB was demonstrated by Kawamoto and colleagues [33]. 

**Figure 8:**
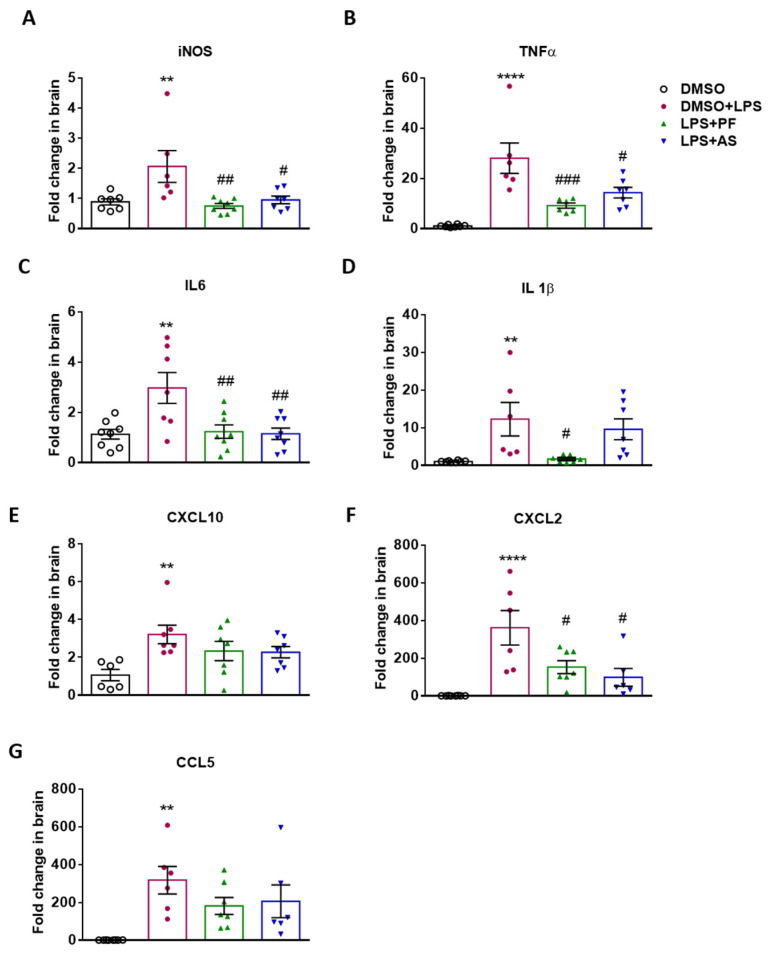
Inhibition of ATX and LPA5 regulates expression of pro-inflammatory genes in LPS-injected C57Bl/6 mice. Mice (n = 6–8 per group) were injected intraperitoneally (i.p.) with DMSO, LPS (5 mg/kg) plus DMSO, with or without PF8380 (‘PF’; 30 mg/kg) or AS2717638 (‘AS’; 10 mg/kg). After 24 h, the animals were sacrificed and the brains were perfused. The right hemisphere from each mouse was processed for RNA isolation and gene expression of (**A**) iNOS, (**B**) TNFα, (**C**) IL6, (**D**) IL-1β, (**E**) CXCL10, (**F**) CXCL2, and (**G**) CCL5 was evaluated by qPCR. Hypoxanthine-guanine phosphoribosyltransferase (HPRT) was used as housekeeping gene. Expression was calculated using the 2−ddCt method. Results are presented as mean values ± SEM of 6–8 mice per group (** *p* < 0.01, **** *p* < 0.0001 compared to DMSO control; # *p* < 0.05, ## *p* < 0.01, ### *p* < 0.001 compared to LPS-treated mice; one-way ANOVA with Bonferroni correction).

Finally, we investigated whether ATX and LPA5 antagonism would affect LPS-induced neuroinflammation in vivo. Using a previously published model of acute LPS exposure [25], the mice were injected with LPS (5 mg/kg), perfused after 24 h, RNA was isolated from one brain hemisphere, and gene expression was analyzed by qPCR. Twenty-four hours following LPS application, the gene expression of iNOS, TNFα, IL6, IL-1β, CXCL10, CXCL2, and CCL5 were significantly upregulated in comparison to DMSO-injected (vehicle) animals (Figure 8). Co-injection of LPS and PF8380 (30 mg/kg) significantly reduced iNOS, TNFα, IL-1β, IL-6, and CXCL2 gene expression. CXCL10 and CCL5 were not regulated by PF8380. Co-injection of LPS with AS2717638 (10 mg/kg) resulted in significantly decreased transcription of iNOS, TNFα, IL6, and CXCL2 in comparison to LPS. IL-1β, CXCL10 and CCL5 showed only a non-significant downward trend. 

**Figure 9:**
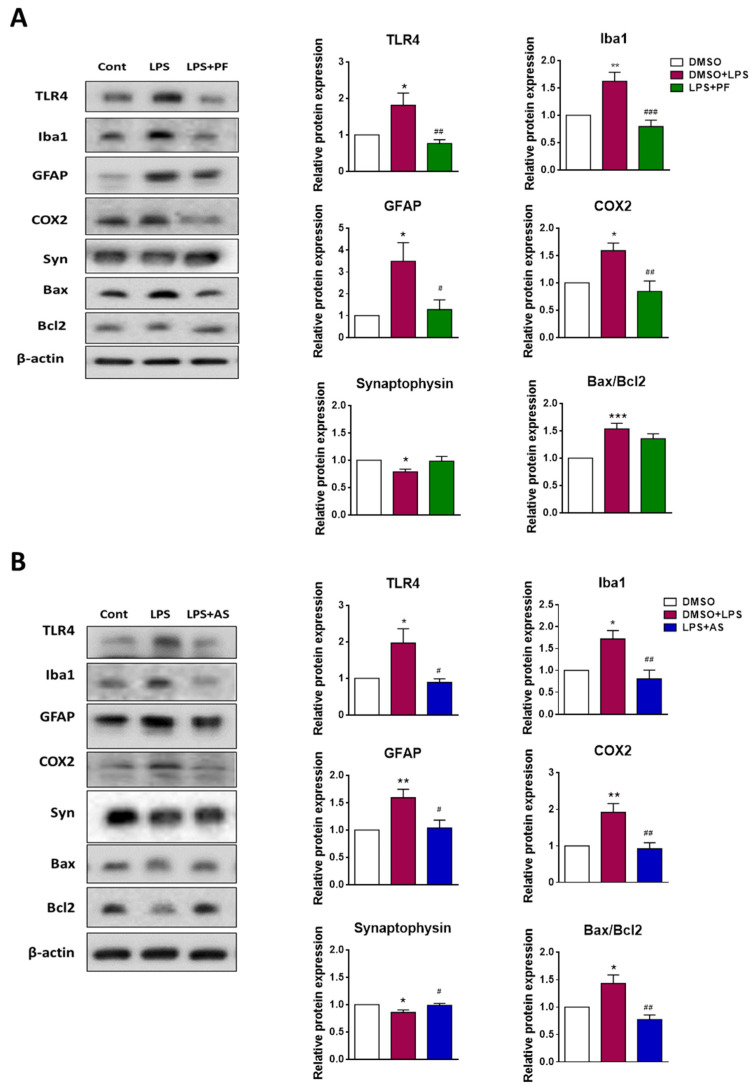
Inhibition of ATX and LPA5 regulates proteins involved in neuroinflammation in LPS-injected C57Bl/6 mice. Mice (n = 6–8 per group) were injected intraperitoneally (i.p.) with DMSO, LPS (5 mg/kg) plus DMSO, with or without (**A**) PF8380 (‘PF’; 30 mg/kg) or (**B**) AS2717638 (‘AS’; 10 mg/kg). After 24 h, the animals were sacrificed and the brains were perfused. The left hemisphere from each mouse was processed for protein analyses by immunoblotting. Protein expression of TLR4, Iba1, GFAP, COX2, synaptophysin, Bax, and Bcl2 were monitored by Western blot analyses. β-actin was used as loading control. One representative blot for each protein is shown. Densitometric evaluation of immunoreactive bands is shown in the bar graphs. Values are expressed as mean ± SEM of 6–8 mice per group (* *p* < 0.05, ** *p* < 0.01, *** *p* < 0.001 compared to DMSO control; # *p* < 0.05, ## *p* < 0.01, ### *p* < 0.001 compared to LPS-treated mice; one-way ANOVA with Bonferroni correction).

The other brain hemisphere of the corresponding animals was processed, to study the expression of a set of proteins that are related to the severity of neuroinflammation. In these experiments, we assessed the expression of immunoreactive TLR4, Iba1 and GFAP (gliosis markers), COX2 (M1 marker), synaptophysin (synaptic integrity marker), Bax (inducer of apoptosis), and Bcl2 (repressor of apoptosis). LPS led to an increase in TLR4, Iba1, GFAP, COX2, and increased the Bax/Bcl2 ratio, while synaptophysin was decreased (Figure 9A). LPS-mediated upregulation of TLR4, Iba1, GFAP, and COX2 were normalized in LPS/PF8380-injected mice (Figure 9A). In a second set of experiments, the animals were injected with DMSO, LPS, or LPS/AS2717638. LPS increased TLR4, Iba1, GFAP, COX-2, and increased the Bax/Bcl2 ratio, while synaptophysin was decreased (Figure 9B). Co-administration of the LPA5 antagonist amended all of the neuroinflammatory parameters that were analyzed during these experiments (Figure 9B).

**Figure 10:**
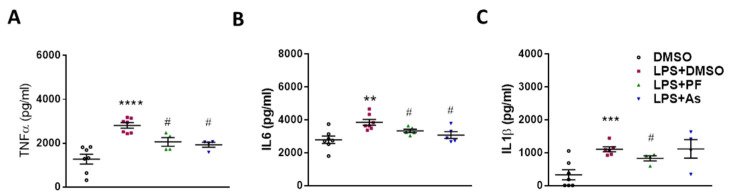
Inhibition of ATX and LPA5 downregulates peripheral cytokine concentrations in LPS-injected C57Bl/6 mice. Mice (n = 6–8 per group) were injected i.p. with DMSO, LPS (5 mg/kg) plus DMSO, with or without PF8380 (‘PF’; 30 mg/kg) or AS2717638 (‘AS’; 10 mg/kg). After 24 h, the animals were sacrificed, blood was collected and processed to obtain serum. Serum was diluted at 1:10 for further analysis. (**A–C**) The concentrations of TNFα, IL6 and IL-1β were quantified using ELISA. Values are expressed as mean ± SEM of 6–8 mice per group **p < 0.01, ***p < 0.001, **** *p* < 0.0001 compared to DMSO control; # *p* < 0.05 compared to LPS-treated mice; one-way ANOVA with Bonferroni correction).

To corroborate that the improvement of the neuroinflammatory conditions in response to PF8380 and AS2717638 is also reflected in the periphery, we performed ELISA measurement in serum. LPS administration significantly increased the serum concentrations of TNFα, IL6, and IL-1β (Figure 10). PF8380 led to a statistically significant reduction in TNFα, IL-6, and IL-1β, while AS2717638 significantly attenuated TNFα and IL-6—but not IL-1β—concentrations.

## 3. Discussion

Studies in experimental animal models revealed that systemic administration of LPS initiates a complex immunological response in the brain, resulting in microglial activation, priming and/or tolerance, memory deficits, and loss of brain synapses and neurons [34]. Therefore, peripheral endotoxemia models are commonly used to induce neuroinflammation. Of note, clinical evidence highlights the close and complex connection between systemic inflammation and neuroinflammation. Zhan and colleagues reported that brain endotoxin levels are elevated two- to three-fold in Alzheimer’s disease, with LPS colocalizing with Aβ1-40/42 in amyloid plaques and around vessels in the AD brain [35]. Studies revealed that the mean blood endotoxin levels are elevated in ALS patients, possibly as a result of gut inflammation and microbiome changes [36]. Also, peripheral diseases that elevate blood endotoxin, such as sepsis and AIDS, are known to lead to neurodegeneration [34]. In mice, a single systemic injection of LPS results in elevated LPA concentrations in the blood and brain, and induces differential regulation of ATX and LPARs in the brain and FACS-sorted microglia [25]. Therefore, we assessed the potential of an ATX and LPA5 inhibitor to ameliorate the neuroinflammatory pathways that are elicited in response to peripheral LPS. For these studies, we used the BV-2 microglia cell line that shows some, but not all, of the features of primary murine microglia [37], and a single i.p. injection of LPS in C57Bl/6 mice. Our study indicates that both ATX and LPA5 antagonism have potential to dampen the LPS-induced neuroinflammatory response in vitro and in vivo. 

The role of the ATX/LPA axis in inflammatory diseases is not entirely clear. In an LPS-induced acute kidney injury mouse model, an LPA injection prior to LPS application provided protection (i.e., reduced urea and creatinine levels) and decreased concentrations of circulating pro-inflammatory cytokines [38]. In the murine EAE model of multiple sclerosis (MS) and in MS patients, the concentrations of several LPA species are reduced, and subsequent deficiency in LPA2 signaling in immune cells promotes disease [39]. Also, in ATX-overexpressing BV-2 microglia, the LPS-induced inflammatory phenotype was less pronounced as compared to wild-type cells [27]. Ciesielska and colleagues reported that the binding of LPA to LPA5 and LPA6 fine tunes the LPS inflammatory response by activating p38, upregulating IL-10, and down-regulating TNFα production in J774 cells [40]. Another study demonstrated a potential anti-inflammatory role of LPA in the LPS-mediated inflammatory response in macrophages, via p38, Akt, and NF-κB [41]. In the LPS-induced acute lung injury mouse model, on the other hand, genetic or pharmacologic targeting of ATX had only minor effects on disease severity [42]. In contrast, there is evidence that a dysregulated ATX/LPA axis (decreased LPC, increased ATX expression and LPA concentration) in acute-on chronic liver failure (ACLF) associates with mortality and systemic infection [43]. The same group reported that LPS stimulates ATX gene expression and increases the inflammatory phenotype of monocytes that are isolated from ACLF patients [43]. In line with this, it was demonstrated that LPS induces a massive increase in ATX mRNA and protein expression via autocrine IFN-γ signaling in monocytic THP-1 cells [44]. In rodent models of neuropathic and inflammatory pain, pharmacological inhibition of LPA synthesis or downstream signaling has beneficial effects [45], and comparable observations were reported in experimental models of demyelination [23], traumatic brain injury [46], experimental autoimmune encephalomyelitis [47], or focal cerebral ischemia [48]. During the present study, pharmacological inhibition of ATX and LPA5 showed beneficial effects using in vitro and in vivo LPS models.

During adulthood, ATX is highly expressed in adipose tissue, and is implicated in the development of metabolic disorders such as the metabolic syndrome and inflammatory diseases [17]. Obesity triggers low-grade inflammation, which is associated with ‘metabolic endotoxemia’, most probably due to the release of low levels of gut-derived LPS [49]. Here, we show, in line with others [27], that LPS modulates ATX and LPAR expression in BV-2 cells. LPS-polarized cells exhibited an increase in ATX protein as well as PLD activity and upregulation of LPAR expression. These findings indicate that in BV-2 cells, LPS could act as a primary inflammatory stimulus that leads to increased LPA synthesis, probably as a secondary, autocrine amplifier of the inflammatory response. In line with such a function, pharmacological inhibition of ATX and LPA5 in LPS-primed BV-2 cells reduced several proinflammatory parameters, including the phosphorylation/activation of proinflammatory transcription factors, TLR4 and COX-2 protein expression, NO production, neurotoxicity, and cyto-/chemokine secretion (Figure 3, Figure 4, Figure 5 and Figure 6).

TLR4 binding of LPS is facilitated in conjunction with LPS binding protein, CD14, and MD-2. As a co-receptor, CD14 sensitizes cells to LPS by transferring LPS molecules to TLR4. Since CD14 transcription and translation is strongly induced in response to LPA [50], LPA could amplify TLR4 signaling induced by LPS. TLR4 activation by LPS triggers the following two consecutive signaling cascades that rely on (intra)cellular receptor localization: plasma membrane-localized TLR4 triggers the Myd88, while internalized, endosomal TLR4 triggers TRIF-dependent signaling [51]. These pathways lead to a synchronized production of pro- and anti-inflammatory mediators. The Myd88 pathway is responsible for COX-2, NOS2, TNFα, and IL-6 production, while the TRIF axis contributes to CCL5 and CXCL10 production. In addition, TLR-4 is involved in canonical activation of the NLRP3 inflammasome, where caspase-1 contributes to Il-1β synthesis. Our studies indicate that PF8380 and AS2717638 are able to attenuate pro-inflammatory effects in LPS-induced inflammation in BV-2 microglia. Western blot analysis revealed suppression of TLR4 and COX-2 in PF8380- and AS2717638-treated BV-2 cells (Figure 4). In line with this, LPA was shown to induce TLR4 expression [52], while AM095, an LPA1 antagonist, suppresses TLR4 expression in mesangial cells [53]. Although both inhibitors that were used during the present study attenuated cyto-/chemokine concentrations in the cellular supernatant of LPS-treated BV-2 cells, the effects were more clear-cut for the CXCL10, CXCL2 and CCL5 chemokines (Figure 5 and Figure 6). These findings could indicate that inhibition of LPA synthesis and/or downstream signaling preferentially attenuates the TLR4-dependent TRIF axis. 

Our in vivo studies further demonstrated the anti-inflammatory potential of PF8380 and AS2717638 in a murine endotoxemia model. Here, it is noteworthy that we have adopted a prophylactic, rather than a therapeutic, treatment regimen. Using a co-administration protocol of LPS and PF8380 or AS2717638, we found that both compounds attenuated iNOS, TNFα, IL-6, and CXCL2 mRNA expression, while IL-1β was only inhibited by PF8380 (Figure 8). On the protein level, both inhibitors reduced LPS-induced TLR4 expression back to baseline levels, and normalized several marker proteins, being indicative of gliosis (Iba1, GFAP), inflammation (COX-2), apoptosis (Bax/Bcl2), or neuronal death (synaptophysin; Figure 9). This is in line with the findings reported by Sapkota and colleagues that demonstrated that TCLPA5 (LPA5 inhibitor) ameliorated microglia activation and cytokine mRNA expression levels in the tMCAO mouse model [48]. Finally, we could show that peripheral cytokine concentrations were also significantly reduced in the peripheral circulation, in response to ATX or LPA5 inhibition (Figure 10). These findings indicate that reduced cytokine concentrations in the periphery could diminish neuroinflammation in inhibitor-treated animals, due to decreased signaling across cytokine receptors at the blood–brain barrier.

LPA effects in a given cell type or organ will depend on its local concentration, which is regulated by synthesis via ATX or degradation by LPPs, the relative abundance of different receptor subtypes, and the presence of potential agonists and/or antagonists [54]. Thus, pharmacological targeting of the ATX/LPA axis likely needs some caution. This is based on findings that both ATX knockout or overexpression are embryonically lethal [18,55,56], demonstrating the requirement for tight control of LPA levels during development. In contrast, in adult mouse life, ATX inhibition using high-dose PF8380 appears to be pharmacologically safe [57], indicating that under certain circumstances ATX represents a ‘druggable’ target. Structural studies revealed that ATX has a tripartite binding site, consisting of the catalytic site, a hydrophobic pocket, and a hydrophobic channel [58]. Based on different binding modes, ATX inhibitors were classified into four groups (type I–IV inhibitors) [59]. PF8380, which was used during the present study, belongs to the type I inhibitors that occupy the catalytic site and mimic binding of LPC. PF8380 is, to the best of our knowledge, not listed in clinical trials yet (https://clinicaltrials.gov/, accessed on 1 July 2021). The type IV ATX inhibitor GLPG1690 (ziritaxestat) was the first ATX antagonist used in humans and showed promising results in a phase 2a randomized placebo-controlled trial to treat idiopathic pulmonary fibrosis [60]. However, only recently, Galapagos NV announced the discontinuation of the ziritaxestat research program, including the ISABELA phase 3 trials (https://www.glpg.com/IPF, accessed on 1 July 2021), since the benefit–risk profile no longer supports continuing the studies (NCT03733444). LPA5, the second pharmacological target studied here, is highly expressed in the spinal cord and dorsal root ganglion [61], and shows high expression in BV-2 and primary murine microglia [30]. LPA5 is associated with inflammatory and neuropathic pain [29], and induces a proinflammatory microglia phenotype [26]. Consequently, the LPA5 antagonist AS2717638 showed broad analgesic effects in several animal pain models [29] and inhibited LPA-mediated proinflammatory polarization of BV-2 cells [26].

In summary, the results from the present study indicate that inhibition of the ATX/LPA5 axis in endotoxemia inhibits neuroinflammation in vitro (BV-2 cells) and in vivo (C57Bl/6 endotoxemia model). Whether co-inhibition of ATX and LPA5 would provide additional therapeutic benefit, due to the inhibited chaperoning function of ATX for LPA delivery to LPA5 (perhaps in an interplay between surface integrins and proteoglycans; [62]), was not experimentally addressed here.

## 4. Materials and Methods

### 4.1. Materials

Cell culture medium RPMI1640, fetal calf serum (FCS), antibiotics, and trypsin were from Invitrogen (MA, USA). Autotaxin inhibitor (PF8380) was from Echelon Biosciences (UT, USA) and the LPA5 antagonist AS2717638 was a gift from Prof. Marc Nazare (Leibniz-Forschungsinstitut für Molekulare Pharmakologie (FMP), Berlin, Germany). Lipopolysaccharide (LPS) from Escherichia coli (O111:B4), 3-(4,5-dimethyl-2-thiazolyl)-2,5-diphenyltetrazolium bromide (MTT), and the phospholipase D activity assay kit were from Sigma-Aldrich (MO, USA). ELISA kits were from Peprotech (NJ, USA). RNeasy lipid tissue mini kit was from Qiagen (Hilden, Germany). Antibodies were from Cayman chemicals (MI, USA), Abgent (CA, USA), Merck Millipore (MA, USA), Fujifilm Wako Chemicals USA Inc (VA, USA) Cell Signaling Technology (MA, USA), Abcam (Cambridge, UK), Santa Cruz Biotechnology Inc. (TX, USA), and Agilent Dako (CA, USA), (as listed in Table 1). Primers were from Qiagen (Germany) and Invitrogen (MA, USA) (listed in Table 2). Total nitric oxide assay kit was from Enzo Life Sciences, Switzerland. Lactate dehydrogenase (LDH) activity kit was from Cayman Chemicals (MI, USA).

### 4.2. BV-2 Microglia

The BV-2 murine microglia cell line was purchased from Banca Biologica e Cell Factory (Genova, Italy). Cells were grown and maintained in RPMI1640 medium supplemented with 10% FCS, 100 units/mL penicillin, 100 µg/mL streptomycin, 1% L-glutamine (stock 200 mM) and cultured in a humidified incubator under 5% CO_2_ and 95% air. The culture medium was changed to fresh medium every 2–3 days. When cells reached confluency, they were split in new flasks or processed for experiments.

### 4.3. CATH.a Neurons

The murine neuronal cell line CATH.a was obtained from ATCC (CRL-11179, (Manassas, VA, USA) and maintained in RPMI1640 medium supplemented with 10% horse serum, 5% FCS, 1% penicillin–streptomycin, 0.4% HEPES, and 0.2% sodium pyruvate at 37 °C in a humified incubator (5% CO_2_, 95% air). When cells reached confluency, they were split into new flasks (subcultivation ratio of 1:4) using 0.12% trypsin without EDTA or used immediately for the experiments.

### 4.4. LPS Treatment of BV-2 Cells

LPS stock solution of 1 mg/mL was prepared in water, and aliquoted and stored at −20 °C. Cells were incubated with LPS 20 ng/mL with or without inhibitors.

### 4.5. Treatment with Pharmacological Antagonists

PF8380, an autotaxin inhibitor, and AS2717638, an LPA5 inhibitor (the IC_50_ values for LPA1-3 are >10 µM; Ref. [29]) was used. The inhibitors were dissolved in DMSO to get stock solutions of 10 mM each and used at the indicated concentrations. DMSO was used as vehicle control. Both inhibitors were without effects on transcription factor phosphorylation and COX-2 under basal conditions in BV-2 cells cultured in the absence of FCS (data not shown).

### 4.6. Immunoblotting

Cells were seeded onto 6-well plates at a density of 1 × 10^5^ per well and serum-starved overnight prior to the experiments. Then, cells were treated with LPS (20 ng/mL) in the absence or presence of PF8380 or AS2717638 for the indicated times and concentrations. DMSO was used as vehicle control. At the end of the time points, the medium was removed and cells were washed twice with ice-cold PBS. The cells were lysed in RIPA buffer (50 mM Tris-HCl pH 7.4, 1% NP-40, 150 mM NaCl, 1 mM Na_3_VO_4_, 1 mM NaF, 1 mM EDTA) containing protease inhibitors (Sigma, Missouri, USA; aprotinin, leupeptin, pepstatin: 1 μg/mL each), 10 μM PMSF and phosphatase inhibitor cocktail (Thermo Scientific, Waltham, MA, USA), scraped and centrifuged at 13,000 rpm for 10 min. Protein content was determined using the BCA kit (Thermo Scientific) with BSA as standard. Then, 50 µg of total protein was separated on 10% SDS-PAGE gels and transferred to polyvinylidene difluoride membranes using electrophoretic transfer (Bio-Rad, Berkeley, CA, USA). Membranes were blocked with 5% *w*/*v* low-fat milk in TBST and incubated with primary antibodies overnight at 4 °C. After removal of primary antibodies, the membranes were washed for 30 min in TBST and incubated for 2 h at RT with HRP-conjugated secondary antibodies (anti-rabbit 1:5000; anti mouse 1:5000), followed by washes with TBST for 1 h. Immunoreactive bands were visualized using chemiluminescence HRP substrate development, ECL or ECL plus reagents (Thermo Scientific) and the Bio-Rad ChemiDoc MP imaging system (Bio-Rad, Vienna, Austria). When necessary, the membranes were cut, stripped (70 μL β-mercaptoethanol in 10 mL 60 mM Tris/2% SDS buffer, pH 6.8; 50 °C for 20 min) and re-probed. Anti-β-actin (1:5000) was used as loading control. 

### 4.7. MTT Assay

The mitochondrial-dependent reduction in MTT to formazan was used to measure cellular metabolic activity. Briefly, BV-2 cells were seeded at 1 × 10^4^ cells per well in a 48-well plate. Following overnight serum starvation, cells were treated with PF8380 and AS2717638 for indicated concentration and time periods. At the end of the treatment, MTT was added to the final concentration of 0.5 mg/mL and incubated for 30 min at 37 °C under standard conditions. Then, 200 µL of lysis buffer (isopropanol/1 M HCl (25:1, *v/v*)) was added with vigorous shaking (1200 rpm, 15 min). Further, 100 µL of it was transferred to a 96-well plate. Absorbance was measured at 570 nm on Victor 1420 multilabel counter (Wallac, Turku, Finland) and corrected for background absorption (650 nm).

### 4.8. PLD Activity

Autotaxin belongs to the ENPP family of enzymes and displays phospholipase D (PLD) activity. PLD activity was measured using phospholipase D assay kit (Sigma, Missouri, USA), according to manufacturer’s instructions. Briefly, BV-2 cells were seeded onto 6-well plates at a density of 1 × 10^5^ cells/well and serum-starved overnight prior to experiment. Then, cells were treated with LPS (20 ng/mL) in absence or presence of the indicated concentrations of PF8380 for the indicated time periods. DMSO was used as vehicle control. At the end of the time points, the medium was collected. Then, 10 µL of the supernatant was mixed with 90 µL of the master reaction. After 10 min, initial absorbance, (A_570_) _initial_, was measured at 570 nm. The plate was incubated at room temperature for 20 min and absorbance was measured again to determine (A_570_) _final_. One unit of PLD catalyzes the formation of 1 µmole choline per minute under the assay condition, and is calculated as follows:

Sample PLD activity = (B/(∆T × V)) × D

where the following applies:

B = amount of choline in the sample (nmol);

∆T = reaction time (minutes);

V = sample volume added into the reaction well (mL);

D = sample dilution factor.

### 4.9. ELISA

Concentrations of cytokines (IL-1β, TNFα, IL-6) and chemokines (CCL5 (RANTES), CXCL2 (MIP-2), and CXCL10 (IP-10)) were quantified using murine ELISA development kits (Peprotech, NJ, USA). Briefly, BV-2 cells were seeded onto 6-well plates at a density of 1 × 10^5^ per well and serum-starved overnight prior to experiment. Then, cells were treated with DMSO, LPS (20 ng/mL) plus DMSO, and LPS plus PF8380 (10 and 1 µM) or AS2717638 (1 and 0.1 µM) for the indicated time periods. The supernatant was collected and stored at −80 °C until further use. The assays were performed using manufacturer’s instructions. The concentration of cytokines and chemokines were determined using an external standard curve.

### 4.10. Determination of Nitric Oxide (NO)

The accumulated total nitrate levels were measured in the supernatant of cells that were incubated with LPS in the absence or presence of antagonists in serum-free medium using the total nitric oxide assay kit (ENZO Life Sciences, Lausen, Switzerland). This assay is based on the enzymatic conversion of nitrate to nitrite by the enzyme nitrate reductase, followed by the Griess reaction to form a colored azo-dye product. The samples were processed according to manufacturer’s protocol. A standard curve was generated in the range between 0 and 100 μM using nitrate as standard. The total nitrate concentration per sample was determined using external calibration.

### 4.11. LDH Release from CATH.a Cells (Neurotoxicity Assay)

CATH.a cells were seeded in 96-well plates (1 × 10^5^ cells per well) and allowed to adhere. Following overnight serum starvation, the cells were incubated in the presence of microglia-conditioned medium. Three wells containing only medium without cells were used as background control. As a positive control, cells were incubated with the LDH-positive control solution (100% release). In order to measure maximum and spontaneous release, cells were incubated with 10% Triton X-100 and assay buffer, respectively. Cells were kept at 37 °C/5% CO_2_ for 24 h and then the plate was centrifuged at 500× *g* for 5 min. One hundred microliters of the supernatants were transferred to a new 96-well plate, and 100 μL of LDH reaction solution was added to each well. The plate was incubated at 37 °C/5% CO_2_ for 30 min under gentle shaking and the absorbance at 490 nm was measured using a plate reader.

### 4.12. Acute Model of Neuroinflammation in C57BL/6J Mice

Wild-type C57BL/6J mice (8–10 weeks, 20–30 g) were obtained from the Department of Laboratory Animal Science (Himberg, Austria), and housed and bred in a clean environment and a 12 h/12 h light–dark cycle with chow diet and water ad libitum. All animal experiments were approved by the Austrian Federal Ministry of Education, Science and Research (BMWF-66.010/0067-V/3b/2018). All measures were taken to minimize animal suffering and distress.

Male C57BL/6 mice were separated into different experimental groups (n = 6–8 animals per group), as follows:
Group IDMSO control (3.33 mL/kg);Group IILPS (5 mg//kg) in PBS + DMSO;Group IIIPF8380 (30 mg/kg) in DMSO + LPS;Group IVAS2717638 (10 mg/kg) + LPS.

LPS (in the absence or presence of antagonists) was administered by intraperitoneal injections. At 24 h post LPS application, the animals were euthanized, perfused and the brains were harvested. The right hemisphere of the brain was collected in QIAzol lysis reagent (QIAGEN, Hilden, Germany) for RNA isolation and the left brain hemisphere was processed for Western blot analyses. One hundred mg of brain tissue was homogenized with 1 mL of tissue extraction buffer (100 mM Tris pH 7.4, 150 mM NaCl, 1 mM EGTA, 1 mM EDTA, 1% Triton X-100) containing protease inhibitors (aprotinin, leupeptin, pepstatin: 1 μg/mL each), 10 μM PMSF, and phosphatase inhibitors (Thermo Scientific, Vienna, Austria). Brain tissue was homogenized in a Precellys homogenizer and centrifuged at 13,000 rpm for 20 min at 4 °C. The supernatant was collected to obtain soluble protein and its concentrations were determined using the BCA kit (Thermo Scientific). One hundred micrograms of total cell protein was loaded per lane and further processed for Western blot analyses.

Then, 200–300 µL of blood was collected by cardiac puncture. The tubes containing blood samples were kept at room temperature for 1 h and then centrifuged at 2000× *g* for 10 min. Clear supernatant was collected. Serum was diluted 1:10 and used for ELISA for cytokine measurement.

### 4.13. RT-qPCR Analysis

Total RNA from the brain was extracted using the RNeasy lipid tissue mini kit (QIAGEN, Hilden, Germany) according to manufacturer’s protocol and quantified using NanoDrop (Thermo Fisher Scientific, Waltham, MA, USA). RNA was reverse-transcribed by using SuperScript^®^ III reverse transcription kit (Invitrogen, Waltham, MA, USA). Quantitative real-time PCR (qPCR) was performed on Applied Biosystems 7900HT fast real-time PCR system using QuantifastTM SYBR^®^ Green PCR kit (QIAGEN, Hilden, Germany). Relative gene expression levels were normalized to hypoxanthineguanine phosphoribosyltransferase (HPRT) and calculated using ΔΔCT method [63]. Primer sequences are listed in Table 2.

### 4.14. Analysis and Quantitation of PF8380 by LC-MS/MS in C57Bl/6 Mouse Brain

Brain uptake of PF8380 was studied using LC-MS/MS. Briefly, C57BL/6 mice were administered PF8380 (900 µg) dissolved in 450 µL oral formulation vehicle (Echelon), resulting in a dose of 30 mg/kg body weight. The antagonist was administered by gavage to get an indication about oral bioavailability and uptake efficacy across the gastrointestinal epithelium. At the indicated times mice were transcardially perfused with ice-cold PBS under deep anesthesia, and the brains were dissected and snap frozen in liquid N_2_. Brains were homogenized in a BioPulverizer (BioSpec Products, Bartlesville, OK) and tissue homogenates were weighed and extracted using a modified Bligh & Dyer HCl method [32]. External calibration was performed for PF8380 in a concentration range of 0.1–2 µM, LPA species were quantitated using LPA-C17 as internal standard. Quantitation of LPA and PF8380 was conducted by LC-MS/MS. Chromatographic separation was performed on a Phenomenex Kinetex HILIC column (2.1 × 100 mm, 2.6 µm). Detection was performed on a Thermo Orbitrap Velos Pro (Thermo Fisher Scientific Inc., Waltham, MA, USA) hybrid mass spectrometer, using a HESI II probe in negative ionization mode. Automated identification and quantitation of LPA and PF8380 was performed by lipid data analyzer, as previously reported [64].

### 4.15. Statistical Analysis

Data are expressed as mean ± SEM from at least 3 independent experiments unless specified otherwise. Unpaired Student’s *t*-test (two groups), or one-way ANOVA followed by Bonferroni correction (more than two groups) was used for analysis of statistical significance (using the Graph Pad Prism6 package). All values of *p* < 0.05 were considered significant.

## Figures and Tables

**Table 1:** Suppliers and dilution of antibodies used in immunoblotting experiments.

Antibody	Company	Catalogue Number	Dilution
LPA1	Cayman Chemicals	10005280	1:250
LPA2	Abgent	AP6140a	1:250
LPA3	Cayman Chemicals	10004840	1:400
LPA4	Santa Cruz Biotechnology Inc.	sc46021	1:500
LPA5	Merck Millipore	ABT114	1:500
LPA6	Abcam	ab85894	1:500
TLR4	Santa Cruz Biotechnology Inc.	sc293072	1:500
Autotaxin (E-12)	Santa Cruz Biotechnology Inc.	sc374222	1:500
pSTAT1 Ser727	Cell signaling technologies	cs8826	1:1000
STAT1	Cell signaling technologies	cs14994	1:1000
pp65 Ser536	Cell signaling technologies	cs3033	1:500
p65	Cell signaling technologies	cs4764	1:1000
pc-Jun Ser63	Cell signaling technologies	cs9261	1:500
c-Jun	Santa Cruz Biotechnology Inc.	sc1694	1:500
COX2	Cell signaling technologies	cs12282	1:500
GFAP	Cell signaling technologies	cs80788	1:1000
Iba1	Fujifilm Wako Chemicals	019-19741	1:1000
Bax	Cell signaling technologies	cs2772	1:1000
Bcl2 (D17C4)	Cell signaling technologies	cs3498	1:1000
Synaptophysin	Agilent Dako	GA660	1:3000
β-actin	Santa Cruz Biotechnology Inc.	sc47778	1:1000

**Table 2:** Primers used for qPCR experiments during the present study.

Gene	Company	Catalogue Number
iNOS	Qiagen	QT00100275
HPRT	Qiagen	QT00166768
**Gene**	**Company**	**Forward/Reverse Primers**
TNFα	Invitrogen	F: ACTTCGGGGTGATCGGTCCR: GGCTACAGGCTTGTCACTCG
IL6	Invitrogen	F: TGTTCTCTGGGAAATCGTGGAR: CAAGTGCATCATCGTTGTTCAT
IL1β	Invitrogen	F: CTCTCCACCTCAATGGACAGAR: CGTTGCTTGGTTCTCCTTGT
CXCL10	Invitrogen	F: TTCTGCCTCATCCTGCTGR: AGACATCTCTGCTCATCATTC
CXCL2	Invitrogen	F: AGTGAACTGCGCTGTCAATGR: GCCCTTGAGAGTGGCTATGA
CCL5	Invitrogen	F: GCTGCTTTGCCTACCTCTCCR: TCGAGTGACAAACACGACTGC

## Data Availability

Not applicable.

## Abbreviations

ACLF	Acute-on chronic liver failure
ATX	Autotaxin
BBB	Blood–brain barrier
CNS	Central nervous system
COX2	Cyclooxygenase 2
CSF	Cerebrospinal fluid
DMSO	Dimethylsulfoxide
FACS	Fluorescence-activated cell sorting
GFAP	Glial fibrillary acidic protein
IBA1	Ionized calcium binding adaptor molecule 1
JNK	c-Jun N-terminale Kinasen
LC-MS/MS	Liquid chromatography–mass spectrometry/mass spectometry
LDH	Lactate dehydrogenase
LPA	Lysophosphatidic acid
LPAR	Lysophosphatidic acid receptor
LPC	Lysophosphatidyl choline
LPS	Lipopolysaccharide
MS	Multiple sclerosis
MTT	3-(4,5-dimethylthiazol-2-yl)-2,5-diphenyltetrazolium bromide
NO	Nitric oxide
NOS2	Nitric oxide synthase 2
PLD	Phospholipase D
STAT1	Signal transducer and activator of transcription 1
STAT6	Signal transducer and activator of transcription 6
TF	Transcription factor
TLR4	Toll-like receptor 4
TRIF	TIR-domain-containing adapter-inducing interferon-β
